# Relationship between body mass index and cardiovascular metabolic multimorbidity: a systematic review and meta-analysis

**DOI:** 10.3389/fcvm.2025.1568348

**Published:** 2025-06-17

**Authors:** Min Wu, Yanyan Huang, Qinyu Liu

**Affiliations:** ^1^ Department of Nephrology, The People’s Hospital of Yubei District, Chongqing, China; ^2^ Silver Hair Health Management Center, Zhuhai People's Hospital (Zhuhai Clinical Medical College of Jinan University), Zhuhai, Guangdong, China; ^3^ Department of Nursing, Liangjiang New District People’s Hospital, Chongqing, China

**Keywords:** cardiovascular metabolic multimorbidity, BMI, prevalence rate, meta-analysis, correlation

## Abstract

This study aimed to systematically evaluate the association between body mass index (BMI) and cardiovascular metabolic multimorbidity (CMM). Cochrane Library, CINAHL, EMBASE, Web of Science, and PubMed were searched for observational studies examining the relationship between BMI and CMM, with a search timeframe from database inception to May 16, 2024. The Newcastle-Ottawa Scale (NOS) and the Agency for Healthcare Research and Quality (AHRQ) criteria were used to evaluate the quality of included studies. The meta package in R was used to perform a meta-analysis. Subsequently, pooled odds ratios (OR) and their 95% confidence intervals (CIs) were computed. Eleven (11) studies involving 1,771,748 patients were included in this review, and the meta-analysis was conducted based on 10 studies. The meta-analysis revealed that when BMI was analyzed as a categorical variable, both overweight (pooled OR = 3.52, 95% CI: 1.23–10.05) and obesity (pooled OR = 3.68, 95% CI: 2.60–5.20) were significantly associated with CMM. When BMI was treated as a continuous variable, it was also significantly related to CMM (pooled OR = 2.90, 95% CI: 1.99–3.81). Furthermore, obesity was associated with an increased risk of CMM [hazard ratio (HR) = 3.27, 95% CI: 2.44–3.39]. This study highlighted a positive correlation between overweight/obesity and CMM. Consequently, it is essential to enhance health awareness among high-risk populations, such as older adults and those with obesity. Proactive screening for high-risk individuals and increased public education on CMM are recommended. Weight management strategies should be integrated into CMM management plans for obese patients to reduce the incidence of these conditions.

**Systematic Review Registration:**

https://www.crd.york.ac.uk/PROSPERO/, PROSPERO CRD42024602835.

## 1 Introduction

As the population ages and life expectancy increases, multimorbidity has become increasingly prevalent among older adults, posing a growing public health burden (1). The World Health Organization (WHO) defines “multimorbidity” as the simultaneous occurrence of at least two chronic diseases in an individual. Cardiovascular metabolic multimorbidity (CMM) has emerged as a focus of research in recent years. It is defined as the presence of two or more cardiovascular metabolic diseases simultaneously, including diabetes mellitus (DM), coronary artery disease, and stroke (2). Studies have indicated that the synergistic effects of these multiple diseases significantly exceed the impact of individual diseases, increasing the risk of various adverse health outcomes (3). An epidemiological survey in the United States revealed a CMM prevalence of 14.4% (4). A recent cohort study revealed that the all-cause mortality for individuals with CMM is 3.7 to 6.9 times higher compared to those without cardiovascular metabolic diseases. Additionally, individuals aged 60 years with one cardiovascular metabolic disease have a life expectancy shortened by 6 to 10 years compared to those without such diseases, while individuals with CMM can experience a life expectancy reduction of up to 15 years (5). Clearly, CMM not only affects patient recovery but also adversely impacts disease prognosis, increases healthcare burdens, severely affects quality of life, and raises mortality. Therefore, understanding the underlying pathophysiological mechanisms of CMM and strengthening appropriate health management is becoming increasingly important.

Various studies have explored potential factors associated with CMM. Variables included age, sex, lifestyle (6, 7), cognitive impairment (8), frailty (9, 10), depression (11), obesity (12), and chronic pain (13). Among these factors, we have noted a close relationship between obesity and CMM. Research has indicated that higher BMI is associated with an increased incidence of CMM. Obesity raises the risk of dyslipidemia and systemic inflammation, serving as a significant risk factor for the development of cardiovascular metabolic diseases and DM. There is a link between CMM and BMI, but this link needs to be studied in more detail. In order to investigate the relationship between BMI and CMM, we carried out this systematic review and meta-analysis.

## 2 Methods

### 2.1 Search strategy

Following the Preferred Reporting Items for Systematic Reviews and Meta-Analyses (PRISMA) standards (PROSPERO: CRD42024602835), we carried out this systematic review. A computer-based search was performed across five databases (Cochrane Library, CINAHL, EMBASE, Web of Science, and PubMed), covering the timeframe from database inception to May 16, 2024. The literature search employed a combination of MeSH and free-text words to identify relevant studies in each database. The keywords used for the literature search included: (“cardio metabol*” OR “cardio-metabol*” OR “cardiometabol*”) AND (“multimorbidity” OR “multimorbidit*” OR “multi-morbidit*” OR “comorbidit*” OR “co-morbidit*” OR “multiple chronic diseases*”) AND (“body mass index” OR “obesity” OR “obese” OR “overweight” OR “body weight” OR “BMI”), with Supplementary Appendix S1 providing an example of the search strategy used for the search in PubMed (Supplementary Material S1). The manuscript does not contain clinical studies or patient data.

### 2.2 Inclusion criteria

Inclusion criteria: (1) The study population consisted of patients with CMM; (2) Study designs were limited to observational studies, including case-control studies, cross-sectional studies, longitudinal studies, and cohort studies; (3) The exposure factor was obesity, measured by BMI; (4) The outcome was CMM, defined as the simultaneous presence of two or more cardiovascular metabolic diseases, including DM, coronary artery disease, stroke, and hypertension. Relevant patient history was obtained through clinical diagnosis or self-report. It should be noted that one included study defined CMM as the presence of at least one cardiovascular metabolic disease (diabetes, coronary artery disease, or stroke) alongside hypertension (14).

According to the World Health Organization (WHO) definitions, BMI categories were classified as follows: obesity (≥30.0 kg/m^2^), overweight (25.0–29.9 kg/m^2^), healthy weight (18.5–24.9 kg/m^2^), and underweight (<18.5 kg/m^2^). Exclusion criteria: (1) Non-English literature; (2) Case reports; (3) Conference papers or abstracts; (4) Qualitative studies or reviews; (5) Meeting proceedings, comments, editorials, newsletters, and study protocols.

### 2.3 Literature screening

The retrieved studies were imported into Endnote X9, a reference management program. The remaining articles' titles were examined separately by two authors (WM and LQY) to determine their applicability to the review topic after duplicates were eliminated using Endnote X9. The entire texts of studies that either fully or partially satisfied the inclusion criteria were retained, and the same two authors independently assessed and screened them to decide if they should be included. For disagreements between the two, a third author (HYY) was consulted until a consensus was reached. Every study that was retained was examined by every author, and each study's inclusion decisions were made by consensus.

### 2.4 Data extraction and quality assessment

A standardized data table was created to extract and summarize pertinent information about the included studies. The extracted data included the author, country, publication year, study type, age of the study population, sample size, BMI, and definition of CMM. The Newcastle-Ottawa Scale (NOS), which consists of eight elements addressing exposure/outcome, comparability, and selection, was used to evaluate the quality of cohort studies. A study can be awarded up to nine stars based on the criteria, with more stars indicating higher methodological quality. In addition, cross-sectional studies were evaluated via a checklist recommended by the Agency for Healthcare Research and Quality (AHRQ), which includes 11 items with “yes”, “no”, or “unclear” options. Two authors (WM and LQY) independently assessed the methodological quality of the included studies, and any disagreements were discussed with a third author (HYY) until an agreement was reached.

### 2.5 Statistical analysis

Data management, effect size conversion, and calculation of pooled mean effect sizes were completed using the “meta” package in R. When BMI was treated as a categorical variable, patients with normal body weight were used as the reference group, and the odds ratios (ORs) for underweight, overweight, and obese CMM patients were combined. The pooled OR was calculated either from reported OR values or from converted OR values based on published data. A forest plot was then generated. When BMI was treated as a continuous variable, the mean difference was chosen as the effect measure, and its 95% confidence interval (CI) was calculated. A *P* of <0.05 was considered statistically significant. Using the I-squared statistic (*I*^2^), heterogeneity was appraised. *I*^2^ = 0% indicated no heterogeneity between studies, *I*^2^ < 25% indicated low heterogeneity, 25% ≤ *I*^2^ < 50% indicated moderate heterogeneity, and 50% ≤ *I*^2^ < 75% indicated high heterogeneity. A fixed-effects model was adopted for meta-analysis if no statistical heterogeneity was noted between studies (*P* > 0.10 and *I*^2^ < 50%). Otherwise, a random-effects model was applied. To confirm the robustness of the findings, sensitivity analyses were performed by excluding studies with large effect sizes or a high risk of bias. For studies where relevant data could not be extracted or where standardized effect sizes could not be calculated from the available data, the eligible studies were described.

## 3 Results

### 3.1 Search results

**Figure 1:**
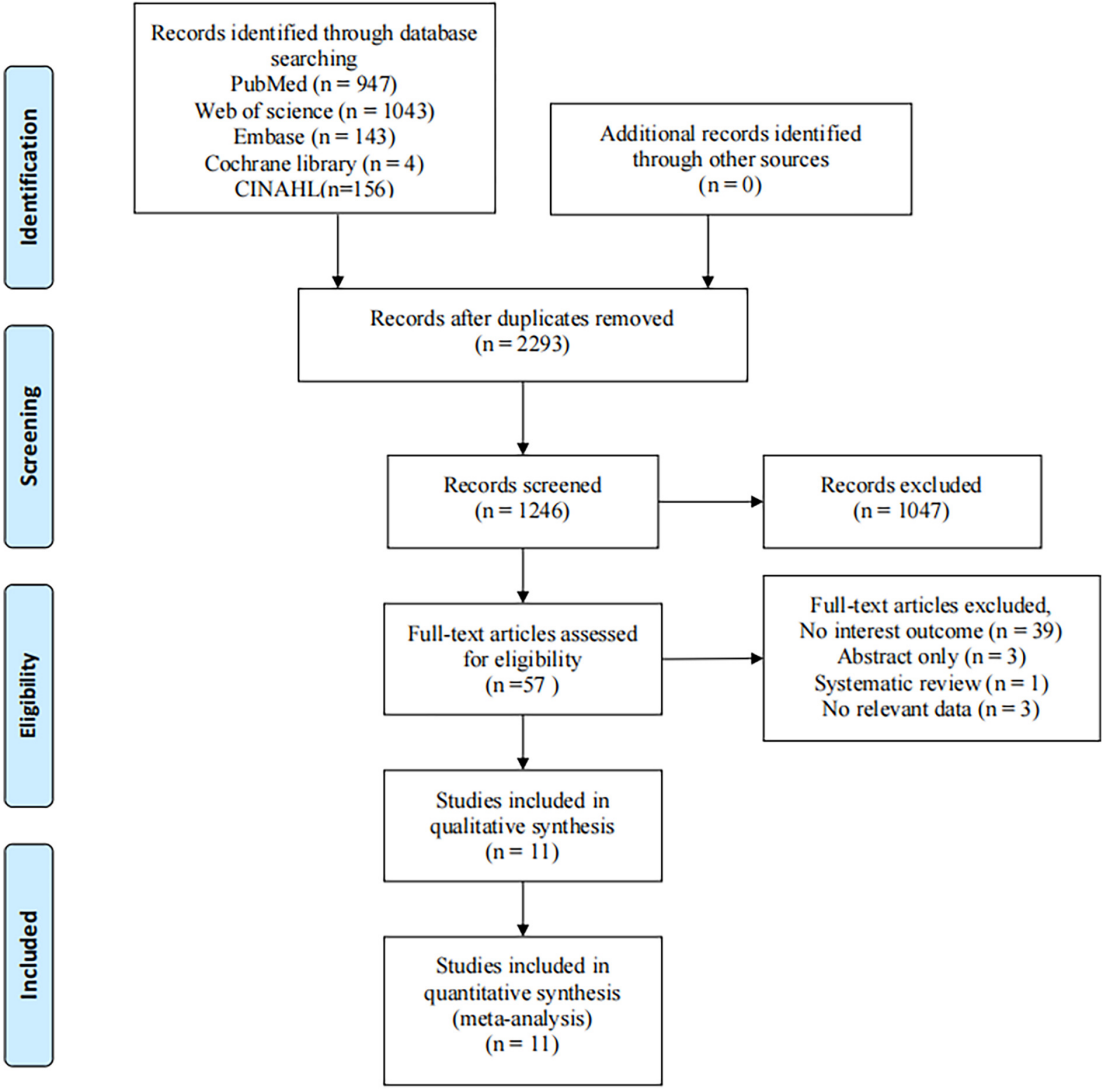
Article selection strategy for the meta-analysis.

A total of 2,293 articles were identified through the search, of which 1,047 were duplicates and removed. After reviewing the titles and abstracts, an additional 1,087 articles were excluded. We further examined 57 articles in detail, and 11 studies published between 2011 and 2024 met the inclusion criteria. Of these, 10 studies were included in the meta-analysis. One cross-sectional study (7) was excluded from the final meta-analysis as it only reported the overall prevalence of CMM without detailing the prevalence among the obese population. The PRISMA flow diagram illustrating the article selection process is provided in Figure 1 below.

### 3.2 Characteristics of the included studies

The characteristics of the included 11 studies (5, 7, 14–22) are summarized in Table 1. These studies were carried out in multiple countries, including China (*n* = 8), the United Kingdom (*n* = 1), South Africa (*n* = 1), and South Korea (*n* = 1). Six studies employed a cross-sectional design, while 5 were cohort studies investigating the association between obesity and CMM. The total sample size across the included studies was 1,759,592 patients, of whom 45,046 were CMM patients, yielding an overall morbidity of 2.56%. Notably, one of the included studies focused on a hypertensive population (14), and 2 studies enrolled patients under the age of 35, while the remaining studies included populations aged ≥35 years.

**Table 1:** Characteristics of the studies included (*n* = 11).

No.	Author	Country	Study type	Research time	Sample source/study population	Age	Sample size	CMM patients	CMM prevalence	Definition of CMM
1	Dong et al, (14)	China	cross-sectional study	2020	the National Basic Public Health Service Project (NBPHS),hypertensive patients	≥35	229,287	9,267	4.04%	At least one cardiometabolic disease with high blood pressure: diabetes, coronary heart disease, or stroke.
2	Chen et al, (15)	China	cross-sectional study	2021	the Electronic Health Management Center in Xin zheng, Henan Province, Central China	≥60	81,532	5,767	7.07%	CM is defined as having two or more of the following three diseases: diabetes, stroke, and coronary heart disease.
3	Lu et al, (16)	China	cohort study	2011–2012	the China Health and Retirement Longitudinal Study (CHARLS)	≥45	10,521	325	3.09%	CM is defined as having two or more of the following three diseases: diabetes, stroke, and heart problems.
4	Qin et al, (17)	China	cross-sectional study	2016	The China Patient-Centered Evaluative Assessment of Cardiac Events Million Persons Project (China-PEACE MPP)	35–75	101,973	11,758	11.53%	The primary outcome of the study was CMM, which was defined as the presence of at least two of the following medical conditions: CHD, stroke, hypertension, and diabetes.
5	Sewpaul et al, (18)	South Africa	cross-sectional study	2011–2012	the South African National Health and Nutrition Examination Survey (SANHANES),	≥15	3,832	500	13.05%	The primary outcome was CM, defined as having any two or more of the following conditions: hypertension, diabetes, stroke and angina.
6	Xia et al, (19)	China	cohort study	2020	The Kailuan cohort	≥ 18	87,512	2,232	2.55%	Cardiometabolic multimorbidity was defined as the coexistence of 2 or 3 CMD events (including the first incidence of myocardial infarction, stroke, and type 2 diabetes).
7	Zhang et al, (20)	China	cohort study	2002–2018	the Chinese Longitudinal Healthy Longevity Survey (CLHLS)	≥60	13,933	975	7.00%	Cardiometabolic multimorbidity was identified as having at least two of the four diseases(hypertension,diabetes, heart disease, stroke).
8	Zhao et al, (21)	China	cohort study	1999–2018	NHANES	≥36	25,994	1,251	4.81%	CMM was defined as two or three of CMDs.
9	Di (1) et al, 2015	England	cohort study	1960–2007	the Emerging Risk Factors Collaboration (general population)	/	689,300	6,931	1.01%	A history of 2 or more of the following: diabetes mellitus, stroke, myocardial infarction (MI).
10	Di (2) et al, 2015	England	cohort study	2006–2010	UK Biobank (general population)	/	4,99,808	3,900	0.78%	A history of 2 or more of the following: diabetes mellitus, stroke, myocardial infarction (MI).
11	Kim et al, (22)	South Korea	cross-sectional study	2014	rural residents in Gyeongju, South Korea,	≥65	932	466	50.00%	CMM pattern included diabetes, dyslipidemia, hypertension, and angina (or myocardial infarction).
12	Zheng et al, (7)	China	cross-sectional study	2020–2022	the Health Management Center of the First Affiliated Hospital of Chongqing Medical University in China	≥45	14,968	1,674	11.18%	we defined CMM as the coexistence of two or more cardiometabolic diseases.

BMI was the primary indicator of overweight or obesity across all studies; although the specific criteria for classifying overweight or obesity varied between studies. For overweight, only one study adhered to the WHO definition (BMI 25.0–29.9 kg/m^2^), while other studies used different thresholds: BMI >24 kg/m^2^ (14), 25–30 kg/m^2^ (20), 24–28 kg/m^2^, 24.0–27.9 kg/m^2^ (19), and 23.0–27.4 kg/m^2^ (23). Regarding the definition of obesity, two studies classified obesity as BMI ≥28 kg/m^2^ (19, 20), two studies used BMI ≥30 kg/m^2^ (18, 21), and one study defined obesity as BMI ≥27.5 kg/m^2^. In addition, the categories “transitioned from non-obese to obese” and “stable obesity” in one study (21) were also combined into the obesity group for statistical analysis (Supplementary Material S2).

### 3.3 Quality assessment of included studies

The quality assessment results of the 6 cross-sectional studies are summarized in Table 2. Of these, 5 studies scored ≥6 points. Five studies did not describe how they assessed or controlled for confounding factors. None of the six studies provided descriptions for item 9 (if applicable, explaining how missing data were handled in the analysis) or item 11 (specifying anticipated follow-up, if any, and the percentage of patients with incomplete data or follow-up). The quality assessment results of the 5 longitudinal studies are summarized in Table 3. All studies scored above 7 points, indicating high quality.

**Table 2:** Agency for research and health quality, AHRQ.

Studies	Item1	Item2	Item3	Item4	Item5	Item6	Item7	Item8	Item9	Item10	Item11	Total score
Dong et al. (14);China	yes	yes	yes	yes	no	yes	yes	yes	unclear	yes	unclear	8
Chen et al. (15); China	yes	yes	yes	yes	no	yes	unclear	unclear	unclear	yes	unclear	6
Qin et al. (17); China	yes	yes	yes	yes	no	yes	unclear	unclear	unclear	yes	unclear	6
Sewpaul et al. (18); South Africa	yes	yes	yes	yes	no	yes	unclear	unclear	unclear	yes	unclear	6
Kim et al. (22); South Korea	yes	yes	yes	yes	no	unclear	unclear	unclear	unclear	yes	unclear	6
Zheng et al. (7); China	yes	yes	yes	yes	unclear	unclear	unclear	unclear	unclear	yes	unclear	5

**Table 3:** Newcastle-Ottawa scale, NOS.

Studies	Item1	Item2	Item3	Item4	Item5	Item6	Item7	Item8	Total score
Lu et al. (16); China	a	a	a	a	a, b	b	a	a	9
Xia et al. (19); China	a	a	a	a	a, b	b	a	a	9
Zhang et al. (20); China	a	a	a	b	a, b	c	a	a	7
Zhao et al. (21); China	a	a	a	a	a, b	c	a	a	8
Di Angelantonio et al. (5); England	a	a	a	a	a, b	b	a	a	9

### 3.4 Meta-analysis results

#### 3.4.1 Relationship between overweight (BMI: 25.0–29.9 kg/m^2^) and CMM

**Figure 2:**
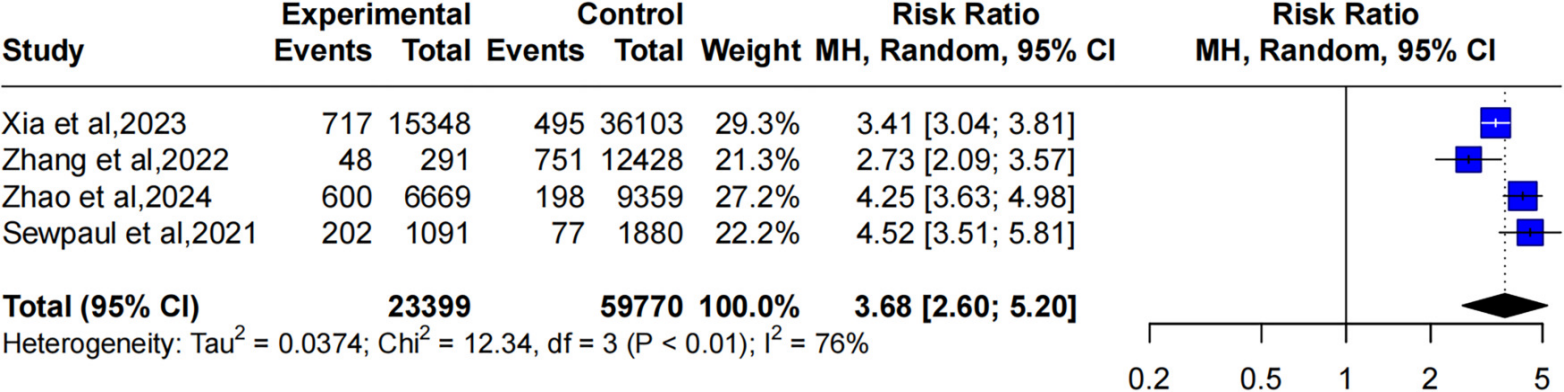
Forest plot comparing the OR of CMM between overweight and healthy weight individuals using a random-effects model.

Five studies reported the relationship between overweight and CMM. The heterogeneity of the included studies was significant (*I*^2^ = 100%, *P* = 0). Thereby, a random-effects model was applied. The pooled results showed that compared to individuals with normal weight, those who were overweight had a higher likelihood of developing CMM (OR: 3.52, 95% CI: 1.23–10.05), as illustrated in Figure 2.

#### 3.4.2 Relationship between obesity (BMI ≥ 30 kg/m^2^) and CMM

**Figure 3:**
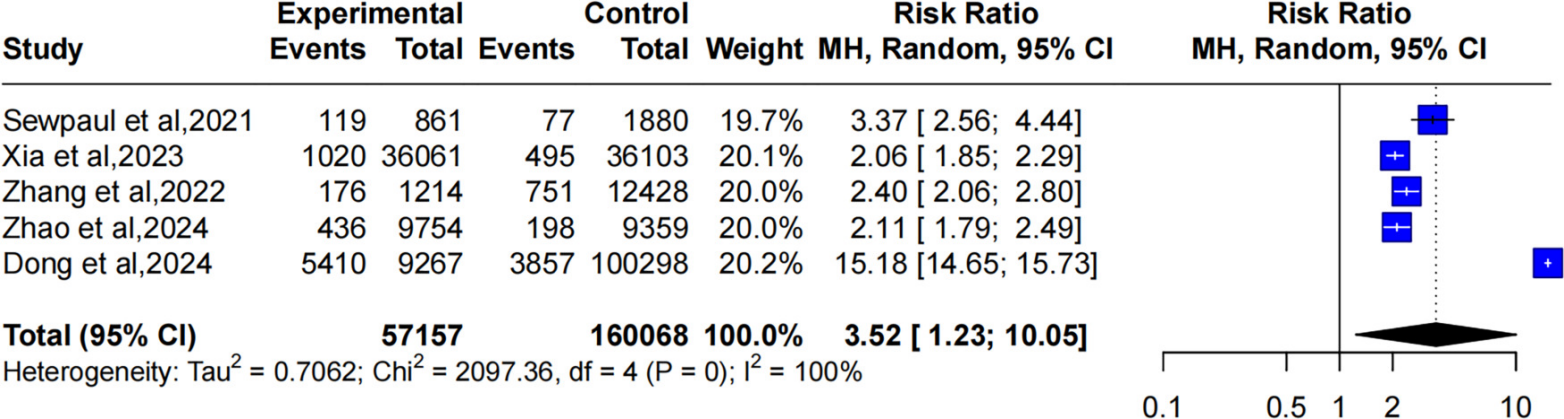
Forest plot comparing the OR of CMM between obese and healthy weight individuals using a random-effects model.

Four studies reported the relationship between obesity and CMM. The heterogeneity of the studies was significant (*I*^2^ = 76%, *P* < 0.01). Hence, a random-effects model was used. The pooled results showed that individuals with obesity had a higher likelihood of developing CMM compared to those with normal weight, and this risk was slightly higher than that for overweight individuals (OR: 3.68, 95% CI: 1.23–10.05), as shown in Figure 3.

#### 3.4.3 Relationship between BMI and CMM

**Figure 4:**
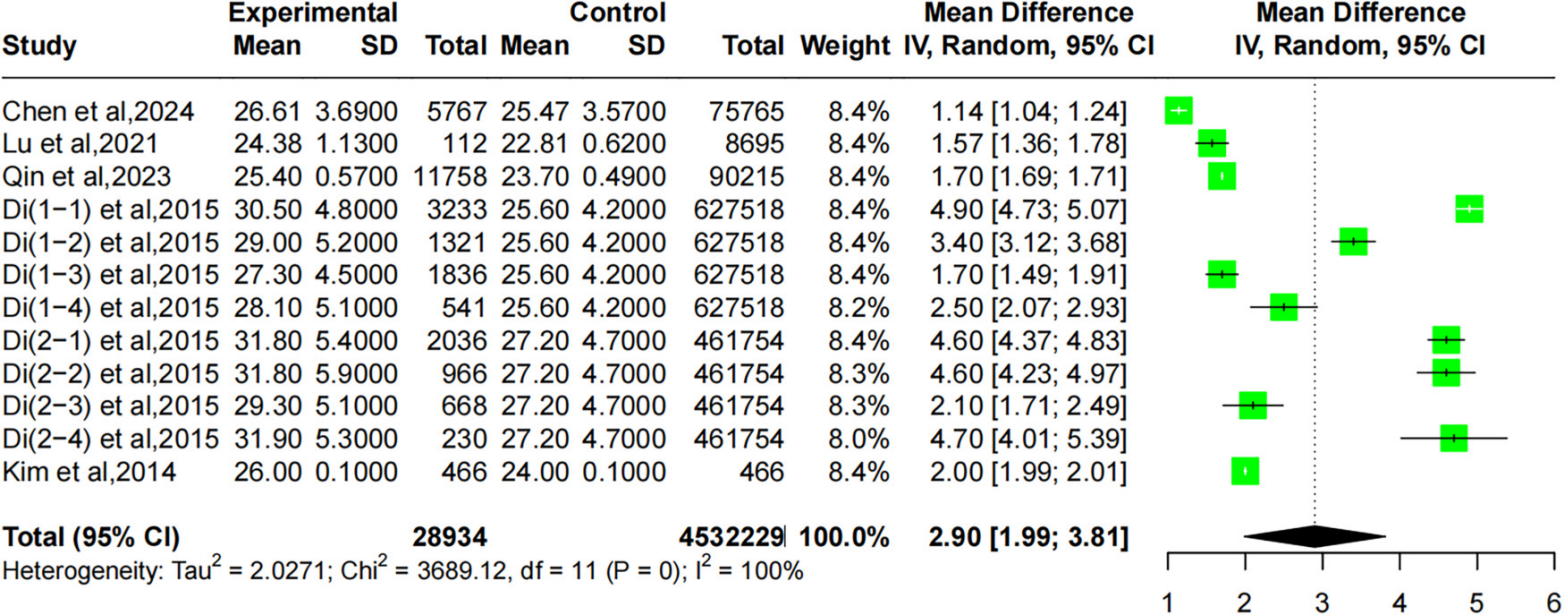
Forest plot comparing the correlation between BMI and CMM using a random-effects model.

Five studies reported the relationship between BMI and CMM. One study by Di included populations from two sample databases, the Emerging Risk Factors Collaboration and the UK Biobank, forming four groups: DM with myocardial infarction, DM with stroke, stroke with myocardial infarction, and individuals with concurrent myocardial infarction, stroke, and DM. The heterogeneity of the included studies was significant (*I*^2^ = 100%, *P* = 0), so a random-effects model was applied. The pooled results showed that the BMI of the CMM population was higher than that of the non-CMM population (MD: 2.90, 95% CI: 1.99–3.81), as illustrated in Figure 4.

#### 3.4.4 Relationship between obesity (BMI ≥ 30 kg/m^2^) and CMM

**Figure 5:**
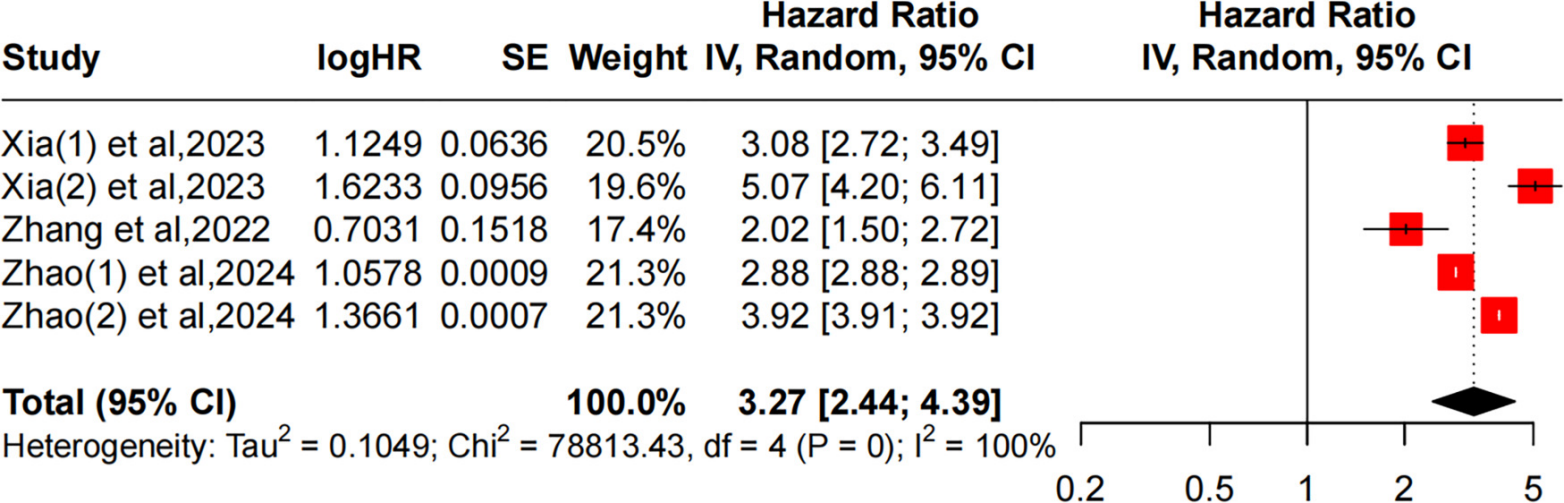
Forest plot comparing the HR of obesity and CMM based on the random-effects model.

Three studies reported the association between CMM and obesity. However, Xia and Zhao extracted two risk ratios related to obesity and CMM. The included studies exhibited significant heterogeneity (*I*^2^ = 100%, *P* = 0), and therefore a random-effects model was applied. The pooled results revealed that the prevalence of CMM in the obese population was 3.27 times that of the normal population (OR: 3.27, 95% CI: 2.44–4.39), as presented in Figure 5.

#### 3.4.5 Sensitivity analysis and subgroup analysis

The sensitivity analysis, which excluded studies with large effect sizes or a high risk of bias, showed no changes in the meta-analysis results, indicating the robustness of the findings. Additionally, a subgroup analysis based on age was conducted, but the heterogeneity remained similar to that of the overall analysis (see Supplementary Materials in the attachment).

#### 3.4.6 Publication bias analysis

Since fewer than 10 studies were included for each outcome, a formal publication bias analysis was not performed.

## 4 Discussion

CMM is becoming a significant public health concern, as it reduces patients' quality of life, increases healthcare costs, and is closely associated with a higher risk of cognitive impairment, frailty, and mortality, imposing substantial economic burdens on individuals, families, and communities (5, 10, 24). The development of CMM is attributed to the direct or indirect interactions between diseases. Studies have shown that having one of ischemic heart disease, stroke, or DM increases the risk of developing the other two diseases (25). Previous research has identified DM as a major risk factor for cardiovascular disease (CVD) (26). Hyperglycemia can disrupt the balance between nitric oxide bioavailability and the accumulation of reactive oxygen species, resulting in endothelial dysfunction, disruption of vascular homeostasis, promotion of inflammatory responses, and thrombus formation, ultimately increasing the risk of CVD (27). On the other hand, prior prospective cohort studies have suggested that CVD can also increase the risk of impaired fasting glucose and DM (28). A well-established cause is that statins, used for lowering cholesterol, can increase the risk of new-onset DM. The possible mechanism is that statins reduce blood lipid levels, affecting the function of voltage-gated calcium channels in pancreatic β-cells, thereby impairing insulin secretion. Additionally, statins can reduce insulin sensitivity in peripheral tissues and impair glucose metabolism, further increasing the risk of DM (29).

To the best of our knowledge, our study is the first to estimate the association between BMI and the risk of CMM. The meta-analysis revealed that when BMI was analyzed as a categorical variable, being overweight (BMI: 25.0–29.9 kg/m^2^) increased the risk of CMM by 3.52 times compared to individuals with a healthy weight, while obesity (BMI ≥ 30 kg/m^2^) increased the risk by 3.68 times. Furthermore, we extracted the risk ratios for CMM in obese populations in three studies, with a pooled result showing that the prevalence of CMM in obese individuals was 3.27 times higher than in those of normal weight. The findings indicated a strong association between both overweight and obesity and the development of CMM, with a higher prevalence of CMM observed in obese individuals compared to those who are overweight. When BMI was analyzed as a continuous variable, the results showed that individuals with CMM had higher BMI levels compared to non-CMM individuals, suggesting that the likelihood of developing CMM increases with BMI. The underlying mechanism may be that early-stage obesity induces a series of metabolic abnormalities, including reduced peripheral glucose uptake, insulin resistance, and glucotoxicity, ultimately leading to elevated blood glucose levels (30). Similarly, obesity can activate the renin-angiotensin system, and alter adipokine and pro-inflammatory cytokine levels, causing hemodynamic changes, microvascular dysfunction, myocardial metabolic abnormalities, atherosclerosis, and calcification, all of which contribute to CVD (31). Obesity is often associated with unhealthy lifestyles, such as sedentary behavior, physical inactivity, unhealthy diets, smoking, and alcohol consumption. Studies have shown that a lack of physical activity can more than double the risk of developing CMM, while unhealthy behaviors like smoking and insufficient sleep also contribute to an increased risk of CMM. A cohort study based on UK Biobank found that, compared to the “very unhealthy” group (characterized by smoking, drinking, poor diet, and lack of physical exercise), the “very healthy” group had a 41% lower risk of CMM among hypertensive patients and a 32%–50% lower risk of specific cardiovascular metabolic diseases, such as metabolic syndrome, stroke, and DM. Among various lifestyle factors, no smoking provided the most protection against CMM. Adopting a combination of healthy lifestyle factors prolonged the life expectancy free of CMM (participants in the “very healthy” group gained up to 6 additional years at age 45). Thus, maintaining a comprehensive healthy lifestyle can help reduce the risk of CMM.

This review has some limitations. Firstly, the majority of the studies employed cross-sectional designs, which do not permit the establishment of a causal relationship between overweight/obesity and CMM. Secondly, 8 of the 11 included studies were conducted in China, and the classification criteria for overweight or obesity differ significantly for Western populations, therefore, caution is needed when generalizing the results. Thirdly, this review does not clarify the biological pathways linking obesity to CMM. To better understand this connection and inform targeted weight management strategies, additional research is essential to uncover the underlying mechanisms. Fourthly, although a comprehensive search was conducted in five English-language databases, some relevant studies may have been missed. Finally, due to the limited number of the included studies, subgroup analyses of predictive factors were not performed. Future studies with more comprehensive data are needed to validate our findings.

## 5 Conclusion

In conclusion, this systematic review and meta-analysis highlights a positive correlation between overweight/obesity and CMM. Therefore, it is essential to raise health awareness among high-risk populations such as older adults and individuals with obesity, actively screen these populations, and enhance education on CMM to help reduce the incidence of the disease.

## Data Availability

The original contributions presented in the study are included in the article/Supplementary Material, further inquiries can be directed to the corresponding author.

## Supplementary material

Supplementary Figure S1 Forest plot comparing the HR of obesity and CMM using a random-effects model.

Supplementary Material S2 Data extraction.
