# Supplementary material for: Relationship between body mass index and cardiovascular metabolic multimorbidity: a systematic review and meta-analysis
Source: Front Cardiovasc Med. 2025 Jun 17;12:1568348. doi: 10.3389/fcvm.2025.1568348 (PMC12211859; doi:10.3389/fcvm.2025.1568348)
Supplement: Supplementary Figure S1 — Forest plot comparing the HR of obesity and CMM using a random-effects model. [file Datasheet1.docx]

**Supplementary Material 1 Search strategy**

**CINHL**


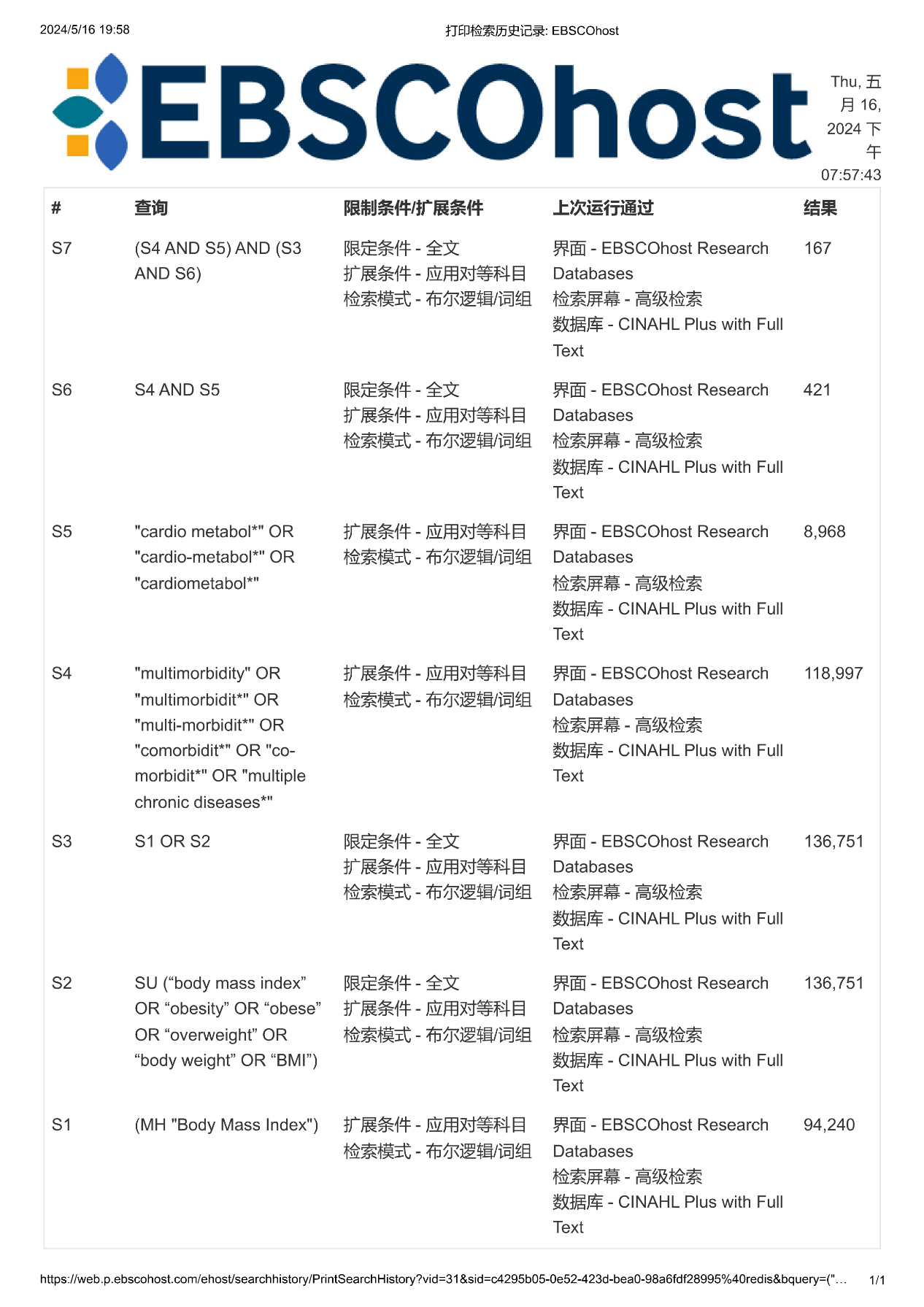


**EMBASE**

| No. | Query | Results | Date |
| --- | --- | --- | --- |
| #9 | #3 AND #8 | 134 | 16-May-24 |
| #8 | #6 AND #7 | 394 | 16-May-24 |
| #7 | 'cardio metabol*':ab,ti OR cardiometabol*:ab,ti | 33655 | 16-May-24 |
| #6 | #4 OR #5 | 15413 | 16-May-24 |
| #5 | multimorbidity:ab,ti OR multimorbidit:ab,ti OR 'multi morbidit':ab,ti OR comorbidit:ab,ti OR 'co morbidit':ab,ti OR 'multiple chronic diseases':ab,ti | 12020 | 16-May-24 |
| #4 | 'multiple chronic conditions'/exp | 9724 | 16-May-24 |
| #3 | #1 OR #2 | 759736 | 16-May-24 |
| #2 | 'body mass index':ab,ti OR obesity:ab,ti OR obese:ab,ti OR overweight:ab,ti OR 'body weight':ab,ti OR bmi:ab,ti | 1280205 | 16-May-24 |
| #1 | 'body mass'/exp OR 'body mass' | 758548 | 16-May-24 |

**PubMed**

| Search number | Query | Results |
| --- | --- | --- |
| 9 | ((Body Mass Index[MeSH Terms]) OR ("body mass index"[Title/Abstract] OR "obesity"[Title/Abstract] OR "obese"[Title/Abstract] OR "overweight"[Title/Abstract] OR "body weight"[Title/Abstract] OR "BMI"[Title/Abstract])) AND ((("Multimorbidity"[Mesh]) OR ("multimorbidity"[Title/Abstract] OR "multimorbidit*"[Title/Abstract] OR "multi-morbidit*"[Title/Abstract] OR "comorbidit*"[Title/Abstract] OR "co-morbidit*"[Title/Abstract] OR "multiple chronic diseases*"[Title/Abstract])) AND ("cardio metabol*"[Title/Abstract] OR "cardio-metabol*"[Title/Abstract] OR "cardiometabol*"[Title/Abstract])) | 947 |
| 8 | (("Multimorbidity"[Mesh]) OR ("multimorbidity"[Title/Abstract] OR "multimorbidit*"[Title/Abstract] OR "multi-morbidit*"[Title/Abstract] OR "comorbidit*"[Title/Abstract] OR "co-morbidit*"[Title/Abstract] OR "multiple chronic diseases*"[Title/Abstract])) AND ("cardio metabol*"[Title/Abstract] OR "cardio-metabol*"[Title/Abstract] OR "cardiometabol*"[Title/Abstract]) | 1,743 |
| 7 | "cardio metabol*"[Title/Abstract] OR "cardio-metabol*"[Title/Abstract] OR "cardiometabol*"[Title/Abstract] | 24,187 |
| 6 | ("Multimorbidity"[Mesh]) OR ("multimorbidity"[Title/Abstract] OR "multimorbidit*"[Title/Abstract] OR "multi-morbidit*"[Title/Abstract] OR "comorbidit*"[Title/Abstract] OR "co-morbidit*"[Title/Abstract] OR "multiple chronic diseases*"[Title/Abstract]) | 240,873 |
| 5 | "multimorbidity"[Title/Abstract] OR "multimorbidit*"[Title/Abstract] OR "multi-morbidit*"[Title/Abstract] OR "comorbidit*"[Title/Abstract] OR "co-morbidit*"[Title/Abstract] OR "multiple chronic diseases*"[Title/Abstract] | 240,567 |
| 4 | "Multimorbidity"[Mesh] | 3,266 |
| 3 | (Body Mass Index[MeSH Terms]) OR ("body mass index"[Title/Abstract] OR "obesity"[Title/Abstract] OR "obese"[Title/Abstract] OR "overweight"[Title/Abstract] OR "body weight"[Title/Abstract] OR "BMI"[Title/Abstract]) | 864,521 |
| 2 | "body mass index"[Title/Abstract] OR "obesity"[Title/Abstract] OR "obese"[Title/Abstract] OR "overweight"[Title/Abstract] OR "body weight"[Title/Abstract] OR "BMI"[Title/Abstract] | 838,359 |
| 1 | Body Mass Index[MeSH Terms] | 152,719 |

**Wos**

| Entitlements | # | Search Query | Database | Results | Date Run |
| --- | --- | --- | --- | --- | --- |
| - WOS.IC: 1993 to 2024 - WOS.CCR: 1985 to 2024 - WOS.SCI: 1975 to 2024 - WOS.AHCI: 1975 to 2024 - WOS.BHCI: 2005 to 2024 - WOS.BSCI: 2005 to 2024 - WOS.ESCI: 2019 to 2024 - WOS.ISTP: 1990 to 2024 - WOS.SSCI: 1965 to 2024 - WOS.ISSHP: 1990 to 2024 | 1 | TS=(“body mass index” OR “obesity” OR “obese” OR “overweight” OR “body weight” OR “BMI”) | Web of Science Core Collection | 995982 | Thu May 16 2024 19:15:44 GMT+0800 (香港标准时间) |
| - WOS.IC: 1993 to 2024 - WOS.CCR: 1985 to 2024 - WOS.SCI: 1975 to 2024 - WOS.AHCI: 1975 to 2024 - WOS.BHCI: 2005 to 2024 - WOS.BSCI: 2005 to 2024 - WOS.ESCI: 2019 to 2024 - WOS.ISTP: 1990 to 2024 - WOS.SSCI: 1965 to 2024 - WOS.ISSHP: 1990 to 2024 | 2 | (TS=("cardio metabol*" OR "cardio-metabol*" OR "cardiometabol*")) AND TS=("multimorbidity" OR "multimorbidit*" OR "multi-morbidit*" OR "comorbidit*" OR "co-morbidit*" OR "multiple chronic diseases*") | Web of Science Core Collection | 1846 | Thu May 16 2024 19:16:51 GMT+0800 (香港标准时间) |
| - WOS.IC: 1993 to 2024 - WOS.CCR: 1985 to 2024 - WOS.SCI: 1975 to 2024 - WOS.AHCI: 1975 to 2024 - WOS.BHCI: 2005 to 2024 - WOS.BSCI: 2005 to 2024 - WOS.ESCI: 2019 to 2024 - WOS.ISTP: 1990 to 2024 - WOS.SSCI: 1965 to 2024 - WOS.ISSHP: 1990 to 2024 | 3 | #1 AND #2 | Web of Science Core Collection | 1043 | Thu May 16 2024 19:17:03 GMT+0800 (香港标准时间) |

**Cochrane Library**

Search Name:

Date Run: 16/05/2024 13:34:59

Comment:

ID Search Hits

#1 MeSH descriptor: [Body Mass Index] this term only 14024

#2 (“body mass index” OR “obesity” OR “obese” OR “overweight” OR “body weight” OR “BMI”):ti,ab,kw (Word variations have been searched) 160008

#3 #1 OR #2 160008

#4 MeSH descriptor: [Multimorbidity] this term only 154

#5 ("multimorbidity" OR "multimorbidit" OR "multi-morbidit" OR "comorbidit" OR "co-morbidit" OR "multiple chronic diseases"):ti,ab,kw (Word variations have been searched) 917

#6 #4 OR #5 917

#7 ("cardio metabol" OR "cardio-metabol" OR "cardiometabol"):ti,ab,kw (Word variations have been searched) 5200

#8 #6 AND #7 15

#9 #8 AND #3 4
